# The Second of Two One-Year, Multicenter, Open-Label, Repeat-Dose, Phase II Safety Studies of PrabotulinumtoxinA for the Treatment of Moderate to Severe Glabellar Lines in Adult Patients

**DOI:** 10.1093/asj/sjaa382

**Published:** 2021-05-03

**Authors:** Z Paul Lorenc, Jeffrey M Adelglass, Rui L Avelar, Leslie Baumann, Kenneth R Beer, Joel L Cohen, Sue Ellen Cox, Steven H Dayan, Jeffrey S Dover, Jeanine B Downie, Zoe Diana Draelos, Mitchel P Goldman, John E Gross, John H Joseph, Joely Kaufman-Janette, Ronald L Moy, Mark Nestor, Joel Schlessinger, Stacy R Smith, Robert A Weiss

## Abstract

**Background:**

PrabotulinumtoxinA is a 900-kDa botulinum toxin type A produced by *Clostridium botulinum*.

**Objectives:**

The authors sought to investigate the safety of prabotulinumtoxinA for treatment of glabellar lines.

**Methods:**

This was a multicenter, open-label, repeat-dose, 1-year phase II safety study. Adults with moderate to severe glabellar lines at maximum frown, as independently assessed by both investigator and patient on the validated 4-point photonumeric Glabellar Line Scale (0 = no lines, 1 = mild, 2 = moderate, 3 = severe), were enrolled. On day 0, patients received an initial treatment (IT) of 20 U prabotulinumtoxinA (4 U/0.1 mL final vacuum-dried formulation injected into 5 glabellar sites). On and after day 90, patients received a repeat treatment (RT) if their Glabellar Line Scale score was ≥2 at maximum frown by investigator assessment. Safety outcomes were evaluated throughout the study.

**Results:**

The 570 study patients received a median total dose of 60 U, that is, 3 treatments. Sixty-one patients (10.7%) experienced adverse events (AEs) assessed as possibly study drug related; 6.5% experienced study drug–related AEs after the IT. With each RT, progressively lower percentages of patients experienced study drug–related AEs. Eight patients (1.4%) experienced study drug–related AEs of special interest: 5 experienced eyelid ptosis (0.9%), 3 eyebrow ptosis (0.5%), 1 blepharospasm (0.2%), and 1 blurred vision (0.2%). Seven patients (1.2%) experienced serious AEs, but none were study drug related. A total of 4060 serum samples were tested for antibotulinum toxin antibodies; no seroconversion was observed.

**Conclusions:**

The safety of RTs of 20 U of prabotulinumtoxinA for moderate to severe glabellar lines was confirmed in this second phase II study based on a broad range of outcomes.

**Level of Evidence: 2:**

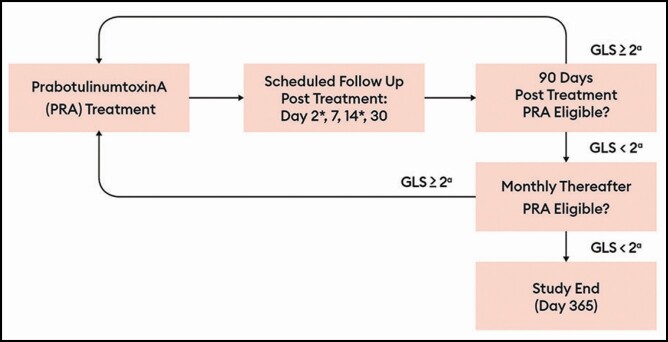

PrabotulinumtoxinA is a new 900-kDa botulinum toxin type A preparation produced by *Clostridium botulinum* first developed by Daewoong Pharmaceutical Co., Ltd. of Seoul, South Korea. It was licensed to Evolus, Inc. of Newport Beach, CA, and is marketed as Jeuveau in the United States. Evidence that an early formulation of prabotulinumtoxinA was both safe and effective for the treatment of moderate to severe glabellar lines in adult patients, and non-inferior to onabotulinumtoxinA (Botox Cosmetic, Allergan, Irvine, CA), was first established in a 268-patient, randomized, double-blind, phase III comparator study conducted in South Korea.^1^

In its final commercial formulation, prabotulinumtoxinA is vacuum-dried; excipients include 0.5 mg Human Serum Albumin (HSA) and 0.9 mg NaCl per 100-U vial. Results from 2 identical multicenter, placebo-controlled, phase III clinical trials (EV-001, n = 330; EV-002, n = 324) conducted in the United States confirmed the efficacy and safety of a single treatment of 20 U of prabotulinumtoxinA (final formulation) for the treatment of moderate to severe glabellar lines in adult patients.^2^ Results from a multicenter, active- and placebo-controlled, phase III clinical trial (EVB-003, n = 540) conducted in Europe and Canada confirmed that a single dose of 20 U prabotulinumtoxinA (final formulation) was both well-tolerated and non-inferior to 20 U onabotulinumtoxinA for the treatment of moderate to severe glabellar lines in adult patients who also felt their glabellar lines had an important psychological impact.^3^ In all 3 of these single-dose studies, the safety endpoints examined included the extent of exposure, total adverse events (AEs), common AEs, serious AEs, AEs of special interest (AESI) as defined by the US Food and Drug Administration (FDA),^4^ study drug–related AEs, vital signs, physical examination, and concomitant medications; in the US EV-001 and EV-002 studies, electrocardiogram and laboratory (hematology, chemistry, urinalysis, serum antibotulinum toxin antibodies) testing were also performed.

The current study, EV-006, was undertaken to investigate the safety of repeat treatments (RTs) of 20 U of prabotulinumtoxinA (final formulation) administered over the course of 1 year for moderate to severe glabellar lines associated with corrugator and/or procerus muscle activity in a large representative US adult population. Safety endpoints examined were comprehensive and identical to those itemized above for the US pivotal EV-001 and EV-002 studies. All efficacy endpoints were exploratory in nature.

## METHODS

### Study Design and Conduct

This study was a prospectively designed, multicenter, open-label (ie, non-blinded), non-randomized, long-term, repeat-dose study in which all patients received active treatment. It was primarily designed to collect long-term safety data related to repeat dosing of prabotulinumtoxinA in a large representative patient population over a 1-year period.

The EV-006 study was conducted between May 2015 and August 2016 at 18 study centers in the United States. The study protocol and its amendments were approved employing a centralized institutional review board review process by Quorum Review IRB of Seattle, Washington. The study was conducted in accordance with the ethical principles that have their origin in the 1975 Declaration of Helsinki and in compliance with the International Conference on Harmonisation harmonised tripartite guideline E6(R1): Good Clinical Practice. ClinicalTrials.gov Identifier: NCT02428608.

### Patients

Study patients were selected from a population of healthy adults, at least 18 years of age, who had moderate (Glabellar Line Scale [GLS] score = 2) to severe (GLS score = 3) glabellar lines at maximum frown as independently assessed by both the investigator and patient employing the validated 4-point photonumeric GLS (see Figure 1 of Beer et al^2^). The main exclusion criteria included previous treatment with botulinum toxin of any serotype in any area within the last 6 months or any planned treatment during the study period, previous treatment with any facial aesthetic procedure in the glabellar area within the last 12 months, previous insertion of permanent material in the glabellar area, any surgery in the glabellar area or any other planned facial aesthetic procedure during the study, marked facial asymmetry, and presence or history of eyelid and/or eyebrow ptosis. Female patients of childbearing potential were required to have a negative pregnancy test and be willing to utilize an acceptable form of contraception. All patients provided written informed consent prior to entering the study.

**Figure 1.**
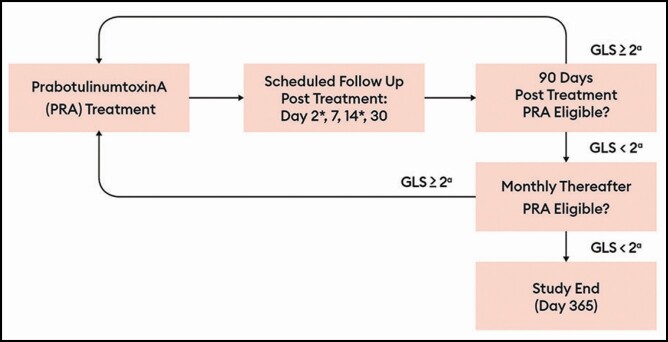
Treatment flowchart. Highlights of the study design included: adverse event assessment at each visit. Dose interval, ≥3 months. Monthly visits after day 90 for ineligible patient to assess for repeat injection eligibility. During repeat injection phase, day 2 and day 14 follow-up were conducted by phone and included a directed questionnaire. No new treatment was offered after day 330. ^a^At maximum frown, by investigator assessment. GLS, Glabellar Line Scale.

### Treatments and Follow-Up

On day 0, eligible patients were treated with 20 U of prabotulinumtoxinA administered as 4 U/0.1 mL injected into each of 5 sites. Investigators administered the study treatment by intramuscular injection. The target injection sites were the midline of the procerus, the inferomedial aspect of each corrugator muscle, and the superior middle aspect of each corrugator at least 1 cm above the bony orbital rim. Topical anesthesia was allowed if determined necessary by investigator and/or patient. After the initial treatment (IT) on day 0, patients were followed-up in the office on days 2, 7, 14, 30, and 90.

On and after day 90 (±7 days), patients were eligible for a RT if their GLS score was ≥2 at maximum frown as judged by the investigator. If a patient did not have a GLS score ≥2, they were followed-up monthly (±14 days) until eligible for RT or until the study ended on day 365. After an RT, the follow-up schedule was similar to the follow-up after the initial injection with the exception that day 2 and day 14 were conducted by telephone call from the investigator’s office instead of an office visit. Patients could have received a maximum of 4 treatments (ie, the IT, and repeat treatments 1, 2 and 3 abbreviated as RT1, RT2, and RT3); they were to be followed-up for a maximum of 365 days from IT. To ensure that there was at least 1 month of follow-up after the last injection, no treatment was to take place after day 330. A schematic of the RT evaluation cycle is presented in Figure 1.

### Assessments

Safety was evaluated by assessing the extent of exposure, AEs, medical histories, physical examination results, vital signs, electrocardiogram and laboratory (hematology, chemistry, urinalysis, and serum antibotulinum toxin antibodies) testing, and concomitant medications. All laboratory and electrocardiogram testing was performed by centralized facilities independent of the sponsor. Hematology, chemistry, and urinalysis testing were performed at screening and end of study/early termination only. General botulinum toxin antibody testing was performed throughout the study at screening, IT days 30 and 90, before each RT, at RT days 30 and 90, and end of study/early termination. In the case of a positive result (ie, evidence of seroconversion), neutralizing antibody testing was to be performed. Electrocardiogram testing was performed at screening, IT day 30, and end of study/early termination.

AEs were collected at each study visit. A directed questionnaire and directed review of systems were employed to help guide the physical examination and ensure that the reporting of AEs—particularly those of special interest—was comprehensive. Of note, the directed questionnaire was administered in person by the investigator or trained investigative site staff in a non-anonymous fashion during the site visit and recorded on paper in the patient’s source documents; the investigator alone was responsible for performing the subsequent directed review of systems and physical examination. AESIs were those 50 events listed in the draft guidance document for industry developed by the US Food and Drug Administration for developing botulinum toxin products for the treatment of upper facial lines.^4^ Examples of AESIs include blurred vision, dysphonia, eyelid ptosis, facial palsy, muscular weakness, and speech disorder.

In addition, efficacy was evaluated at each clinic visit by investigator and patient assessment on the GLS at maximum frown and at rest; investigator and patient assessment on a 5-point Global Aesthetic Improvement Scale (GAIS: 2 = much improved, 1 = improved, 0 = no change, −1 = worse, −2 = much worse); and patient assessment on a 5-point Subject Satisfaction Scale (SSS: 2 = very satisfied, 1 = satisfied, 0 = indifferent, −1 = unsatisfied, −2 = very unsatisfied).

### Outcomes and Statistical Analysis

Analyses were primarily descriptive in nature. Continuous data were summarized by number of patients, mean, standard deviation, median, minimum and maximum; categorical data were summarized by number and percentage of patients. Safety outcomes were reported for the safety population, defined as all patients who received at least 1 dose of prabotulinumtoxinA (ie, the IT on day 0). AEs were coded according to the Medical Dictionary for Regulatory Activities (version 17.0) and grouped by system organ class and preferred term. The incidences of AEs were summarized for each treatment—that is, following the IT, RT1, RT2, or RT3—as frequencies and proportions. The primary safety analysis was the calculation of the proportion of patients with at least 1 AE that occurred from day 0 through day 365.

Exploratory efficacy outcomes were reported for the Response-evaluable population, defined as all patients who received at least 1 dose of prabotulinumtoxinA on day 0 and had at least 1 postbaseline investigator or patient assessment. The only efficacy analysis for which a 95% CI was calculated was the proportion of patients with an improvement from day 0 of 1 point or more (ie, ≥1-point responders) on day 365 on the GLS at rest. Efficacy data were also summarized for various endpoints throughout the study on each of days 2, 7, 14, 30, and 90 and at monthly follow-up visits thereafter. These endpoints included the proportion of patients with a ≥1-point improvement on the GLS at maximum frown, and the distributions of GAIS and SSS scores.

### Sample Size

Approximately 565 patients were planned to be enrolled in the EV-006 study. Assuming a 15% drop-out rate, it was expected that 480 patients would complete the study. This sample size was not based on any specific statistical criteria. Rather, it was based on the need to ensure that there was both a sufficient number of patients in the clinical development program to meet International Conference on Harmonisation guideline requirements for an overall total of over 1500 patients exposed to prabotulinumtoxinA and a sufficient number of patients with exposure to RTs as required by the FDA.

## RESULTS

### Patient Disposition and Demographics

**Figure 2.**
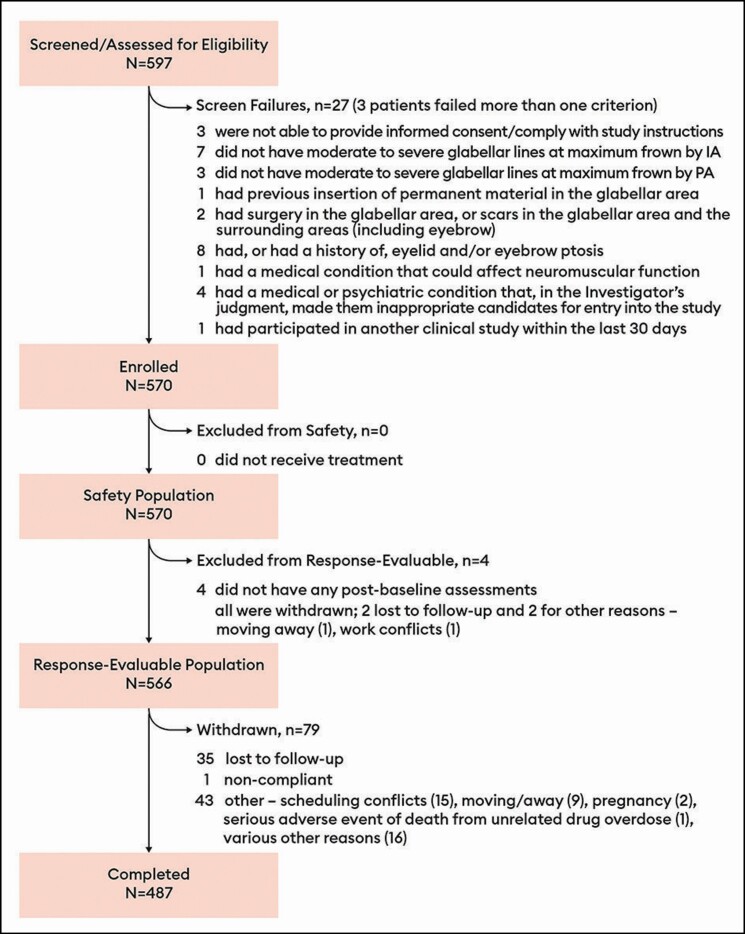
Disposition of all patients: safety and response-evaluable populations. The safety population was all patients who received at least 1 dose of prabotulinumtoxinA, and the response-evaluable population was all patients who received at least 1 dose of prabotulinumtoxinA on day 0 and had at least 1 post-baseline investigator or patient assessment. IA, investigator assessment; PA, patient assessment.

A total of 570 patients were enrolled, received at least the IT of 20 U prabotulinumtoxinA, and formed the safety population (Figure 2). All but 4 of these patients qualified for inclusion in the response-evaluable population. Most patients (487/570, 85.4%) completed the study; most commonly, patients who did not complete did not return and were lost to follow-up (refer to Figure 2 for all other reasons).

Patients had a mean age of 50.8 years (range of 19-77 years) (Table 1). Most patients were younger than 65 years old; 8.9% (51/570) were 65 years of age or older. Most patients (89.5%) were female (510 vs 60 males). Most patients were racially identified as White (76.0%) or were of Hispanic or Latino ethnicity (94/570, 16.5%). The most common Fitzpatrick skin types were III and II, with 36.7% and 28.1% of patients identified with these skin types, respectively. By investigator assessment, 73.3% of patients had severe glabellar lines at maximum frown at baseline; by patient assessment, 81.2% of patients did. By investigator assessment, 88.4% of patients (n = 504) also had evidence of glabellar lines at rest, defined as a baseline GLS score > 0 at rest; by patient assessment, 97.0% (n = 553) did.

**Table 1.** Demographic and Glabellar Line Characteristics at Baseline: Safety Population

Characteristic	PrabotulinumtoxinA (N = 570)	
Age (y)		
Mean ± SD [min, max]	50.8 ± 10.50 [19, 77]	
<65, n (%)	519	(91.1)
≥65, n (%)	51	(8.9)
Sex, n (%)		
Male	60	(10.5)
Female	510	(89.5)
Race, n (%)		
White	433	(76.0)
Black or African American	32	(5.6)
Asian	5	(0.9)
Other^a^	95	(16.7)
Multiple	5	(0.9)
Fitzpatrick skin type,^b^ n (%)		
I	21	(3.7)
II	160	(28.1)
III	209	(36.7)
IV	134	(23.5)
V	27	(4.7)
VI	19	(3.3)
Investigator assessment of glabellar lines on the GLS, n (%)		
At maximum frown		
Moderate	152	(26.7)
Severe	418	(73.3)
At rest		
None	66	(11.6)
Mild	188	(33.0)
Moderate	213	(37.4)
Severe	103	(18.1)
Patient assessment of glabellar lines on the GLS, n (%)		
At maximum frown		
Moderate	107	(18.8)
Severe	463	(81.2)
At rest		
None	17	(3.0)
Mild	109	(19.1)
Moderate	272	(47.7)
Severe	172	(30.2)

GLS, Glabellar Line Scale; SD, standard deviation. ^a^All but 1 patient in the category of “other” identified as Hispanic or Latino. ^b^Type I = always burns, never tans (pale white skin); Type II = usually burns, tans minimally (white skin); Type III = sometimes burns, tans uniformly (cream/light brown skin); Type IV = rarely burns, always tans well (moderate brown skin); Type V = very rarely burns, tans very easily (dark brown skin); Type VI = never burns, deeply pigmented (dark brown to black skin).

## SAFETY

### Extent of Exposure

The 570 patients in the safety population received a mean total dose of 61.0 U of prabotulinumtoxinA (range of 20-80 U) over the course of the 1-year study; the median total dose was 60 U, that is, 3 treatments (Table 2). Of the 487 study completers, 6 patients (1.2%) completed the 1-year study without requiring a RT; at no visit on day 90 or monthly thereafter were these patients assessed by the investigator to have a GLS score at maximum frown of 2 = moderate or 3 = severe. A further 66 patients (13.6%) received a single RT (mean of 199.4 days after the IT; range of 82-330 days), 203 patients (41.7%) received 2 RTs (means of 130.6 and 137.0 days after the initial and first RTs, respectively; ranges of 82-212 days and 84-217 days, respectively), and 212 (43.5%) received 3 RTs (means of 93.9, 96.1, and 99.7 days after the initial, first RT, and second RT, respectively; ranges of 77-145 days, 82-160 days and 65-154 days, respectively) (Tables 2 and 3).

**Table 2.** Extent of Exposure, Summarized by Total Units of PrabotulinumtoxinA Injected and Total Number of Treatments Administered: Safety Population

Total drug administered	Study completers (N = 487)		All patients (N = 570)	
Total dose injected (U), mean ± SD [min, max]	65.5 ± 14.76 [20, 80]		61.0 ± 18.53 [20, 80]	
Median	60		60	
Total treatments administered, n (%)				
1 Treatment (IT only)	6	(1.2)	46	(8.1)
2 Treatments (IT + RT1)	66	(13.6)	93	(16.3)
3 Treatments (IT + RT1 + RT2)	203	(41.7)	217	(38.1)
4 Treatments (IT + RT1 + RT2 + RT3)	212	(43.5)	214	(37.5)
Dose interrupted, n (%)	0	(0.0)	0	(0.0)

IT, initial treatment; RT, repeat treatment.

**Table 3.** Extent of Exposure, Summarized by Number of Days Between PrabotulinumtoxinA

**Total number of days between treatments**	**Mean ± SD [min, max]**	**Median**
Patients who received only 1 treatment (n = 6)		
From IT to end of study	362.8 ± 4.75 [356, 370]	363.0
Patients who received a total of 2 treatments (n = 66)		
Between IT and RT1	199.4 ± 53.02 [82, 330]	190.0
From RT1 to end of study	164.3 ± 55.06 [28, 283]	180.0
Patients who received a total of 3 treatments (n = 203)		
Between IT and RT1	130.6 ± 30.89 [82, 212]	125.0
Between RT1 and RT2	137.0 ± 30.38 [84, 217]	132.0
From RT2 to end of study	94.6 ± 42.45 [27, 203]	91.0
Patients who received a total of 4 treatments (n = 212)		
Between IT and RT1	93.9 ± 11.85 [77, 145]	91.0
Between RT1and RT2	96.1 ± 14.38 [82, 160]	91.0
Between RT2 and RT3	99.7 ± 15.44 [65, 154]	92.0
From RT3 to end of study	74.1 ± 23.17 [25, 140]	81.5

Treatments: study completers only (N** = **487). IT, initial treatment; RT, repeat treatment.

### Adverse Events

A total of 235 patients (235/570, 41.2%) experienced a total of 475 AEs over the course of study (Table 4). Approximately 25% of all patients experienced an AE following the IT, representing 61.3% of all patients (144/235) who experienced an AE at any time during this study. Progressively lower percentages of patients experienced AEs following each RT: 19.3% after RT1, 15.5% after RT2, and 8.9% after RT3. A similar trend was observed for AEs assessed by the investigator as study drug related. In addition, with the exception of the RT3 visit, similar trends were also observed for serious AEs and AESIs (Tables 4 and 5). Note that, overall, few patients experienced these latter types of events, with only 1 patient (0.5%) experiencing a serious AE following RT3 and 3 patients (1.4%) experiencing an AESI following RT3.

**Table 4.** Summary of Treatment-Emergent AEs: Safety Population

Adverse Event Parameter	PrabotulinumtoxinA (N = 570)		
	n/N	(%)	Events, No.
All AEs	235/570	(41.2)	475
Last treatment before onset^a^			
IT	144/570	(25.3)	216
RT1	101/524	(19.3)	147
RT2	67/431	(15.5)	88
RT3	19/214	(8.9)	24
Any serious AE	7/570	(1.2)	8
Last treatment before onset^a^			
IT	3/570	(0.5)	4
RT1	2/524	(0.4)	2
RT2	1/431	(0.2)	1
RT3	1/214	(0.5)	1
Any study drug–related AE	61/570	(10.7)	91
Last treatment before onset^a^			
IT	37/570	(6.5)	46
RT1	19/524	(3.6)	24
RT2	14/431	(3.2)	17
RT3	4/214	(1.9)	4
Any AE leading to study discontinuation	1/570	(0.2)	1
Any AE leading to death	1/570	(0.2)	1
Relationship to study drug			
Not related	174/570	(30.5)	384
Possibly related	30/570	(5.3)	47
Probably related	17/570	(3.0)	23
Definitely related	14/570	(2.5)	21
Severity			
Mild	147/570	(25.8)	333
Moderate	77/570	(13.5)	128
Severe	11/570	(1.9)	14
Frequency			
≥5%	75/570	(13.2)	94
Nervous system disorder, headache^b^	75/570	(13.2)	94

AE, adverse event; IT, initial treatment; n, the number of patients at each level of summarization; RT, repeat treatment. ^a^Percentages are based on the number of patients who received these treatments. ^b^System organ class and preferred term. A patient was counted once in the system organ class if they reported 1 or more events.

**Table 5.** Summary of Treatment-Emergent AESI: Safety Population

AE parameter	PrabotulinumtoxinA (N = 570)								
	All			Study drug related			Not study drug related		
	n/N	(%)	Events	n/N	(%)	Events	n/N	(%)	Events
Any AESI	16/570	(2.8)	21	8/570	(1.4)	11	9/570	(1.6)	10
Last treatment before onset^a^									
IT	7/570	(1.2)	7	4/570	(0.7)	4	3/570	(0.5)	3
RT1	6/524	(1.1)	9	2/524	(0.4)	4	4/524	(0.8)	5
RT2	2/431	(0.5)	2	1/431	(0.2)	1	1/431	(0.2)	1
RT3	3/214	(1.4)	3	2/214	(0.9)	2	1/214	(0.5)	1
Onset, days since last treatment									
Number of events	21			11			10		
Mean ± SD	22.2 ± 27.45			12.4 ± 19.53			33.1 ± 31.62		
Median	9.0			3.0			23.5		
Minimum, maximum	1, 107			1, 66			4, 107		
Duration, d									
Number of events	14			10^b^			4^c^		
Mean ± SD	48.9 ± 94.87			32.5 ± 56.32			89.8 ± 162.37		
Median	11.5			11.5			12.5		
Minimum, maximum	1, 333			1, 183			1, 333		

AESIs were those 50 events potentially suggestive of distant spread of botulinum toxin effects, identified in “Guidance for Industry. Upper Facial Lines: Developing Botulinum Toxin Drug Products.”^4^ One patient had 2 AESIs: 1 was study drug related and 1 was not study drug related. AESI, adverse event of special interest; RT, repeat treatment; SD, standard deviation.

One death (0.2%) was reported in a 43-year-old female who also had breast cancer; this was the only patient who experienced an AE that led to study discontinuation (Table 4). The event was a severe drug overdose (thought to be possibly related to her concomitant medications of alprazolam and temazepan) with an onset 138 days after the IT that was assessed as not related to the study drug. Most AEs (461/475, 97.1%) were mild or moderate in severity (Table 4). Fourteen events (14/475, 2.9%) were severe. These included 2 events of migraine in 1 patient, and 1 each of carotid artery stenosis, headache, overdose, wrist fracture, rash, pain in an extremity, upper abdominal pain, small intestinal obstruction, breast cancer, uterine leiomyoma, anxiety, and drug hypersensitivity. Only the 2 severe events of migraine, which occurred in a single patient with a history of tension headaches (recorded at baseline), were assessed as possibly study drug related; all other severe events were assessed as unrelated.

Seven patients (1.2%) experienced a total of 8 treatment-emergent AEs assessed by the investigator as serious (Table 4): the patient discussed above with breast cancer who died of an overdose, and 1 patient each with squamous cell carcinoma, uterine leiomyoma, colitis, small intestinal obstruction, carotid artery stenosis, and anxiety. No serious event was assessed as study drug related.

Sixty-one patients (10.7%) experienced a total of 91 AEs assessed by the investigator as study drug related (Table 4). Most of the 475 AEs (384/475, 80.8%) reported during the study were assessed as not related to study drug. Altogether, 21 events (4.4%) were assessed as definitely related, 23 (4.8%) as probably related, and 47 (9.9%) as possibly related. Headache was the event most commonly assessed as study drug related; 47 patients (47/570, 8.2%) experienced a headache assessed as either possibly (n = 25), probably (n = 15) or definitely (n = 7) study drug related.

Headache, reported by 13.2% of all patients, was also the most common AE (Table 4). It was the only event reported in 5% or more of patients. By preferred term, a total of 12 other types of AEs occurred in 1% or more of patients (6 or more patients). These included: upper respiratory tract infection (2.8%), sinusitis (2.5%), nasopharyngitis (2.3%), hypertension (1.8%), urinary tract infection (1.8%), contusion (1.6%), bronchitis (1.2%), cough (1.2%), eyelid ptosis (1.2%; see AESI below), acne (1.1%), dermatitis contact (1.1%), and pain in extremity (1.1%).

Sixteen patients (2.8%) experienced a total of 21 AESIs, many of which were assessed as unrelated to study drug (Tables 5 and 6). All AESIs regardless of relatedness were mild in severity; none were assessed as serious, and no patient discontinued the study due to one of these types of events. Eight patients (1.4%) experienced a total of 11 AESIs that were assessed as possibly, probably, or definitely related to study drug (Tables 5 and 6). All 11 events were categorized as eye disorders, including 6 reports of eyelid ptosis in 5 patients (5/570, 0.9%), 3 of eyebrow ptosis (3/570, 0.5%), and 1 each of blepharospasm and blurred vision (each 1/570, 0.2%) (Table 6). Note that, of the 11 events, 4 were reported for a single patient (patient 603024, a 54-year-old female); these included 2 reports of eyelid ptosis (1 event 2 days after the IT that resolved in 66 days, and 1 event 2 days after RT1 that resolved in 22 days), 1 of eyebrow ptosis (2 days after RT1 that resolved in 22 days), and 1 of blepharospasm (8 days after RT1 that resolved in 1 hour). Between 0.2% and 0.9% of patients experienced a study drug–related AESI following any given treatment (Table 5). The median time to onset of study drug–related AESIs was 3 days after the patient’s most recent treatment date, and the median duration was 11.5 days; all resolved. Of particular interest, the 6 study drug–related eyelid ptosis events resolved within 2, 3, 4, 22, 66, and 183 days of onset.

**Table 6.** Treatment-Emergent AESIs by System Organ Class, Preferred Term, Relatedness, Patient Number and Severity: Safety Population

System organ class and preferred term, relationship to study drug (patient no., severity)	PrabotulinumtoxinA (N = 570)		
	n	(%)	Events
All AESIs	16	(2.8)	21
Eye disorders	10	(1.8)	15
Blepharospasm	2	(0.4)	2
Possibly related (603,024, mild)	1	(0.2)	1
Not related (607,001, mild)	1	(0.2)	1
Eyebrow/eyelid ptosis	9	(1.6)	11
Eyelid	7	(1.2)	8
Not related (606,001, mild; 613,009, mild)	2	(0.4)	2
Possibly related (605,011, mild; 606,017, mild)	2	(0.4)	2
Probably related (603,024, mild)	1	(0.2)	1
Definitely related (603,014, mild; 603,024, mild; 603,046, mild)	3	(0.5)	3
Eyebrow	3	(0.5)	3
Possibly related (607,001, mild; 614,015, mild)	2	(0.4)	2
Probably related (603,024, mild)	1	(0.2)	1
Vision blurred	2	(0.4)	2
Not related (606,001, mild)	1	(0.2)	1
Possibly related (607,013, mild)	1	(0.2)	1
Cardiac Disorders	4	(0.7)	4
Sinus bradycardia	2	(0.4)	2
Not related (600,018, mild; 604,020, mild)	2	(0.4)	2
Bradycardia	2	(0.4)	2
Not related (604,008, mild; 604,017, mild)	2	(0.4)	2
Gastrointestinal Disorders	2	(0.4)	2
Dysphagia	2	(0.4)	2
Not related (605,010, mild; 615,020, mild)	2	(0.4)	2

At each level of summarization, a patient was counted once if the patient reported 1 or more events; however, a single patient may be represented at more than 1 level of summarization. AEs were coded employing Medical Dictionary for Regulatory Activities Version 17.0. AEs of special interest were those 50 events potentially suggestive of distant spread of botulinum toxin effects, identified in “Guidance for Industry. Upper Facial Lines: Developing Botulinum Toxin Drug Products.”^4^ AESI, adverse event of special interest; AE, adverse event; n = the number of patients at each level of summarization.

Of the 51 patients (51/570, 8.9%) who were 65 years of age or older, 20 patients (20/51, 39.2%) experienced AEs. One of the 8 serious events (squamous cell carcinoma) and 2 AESI (1 mild bradycardia, 1 mild aponeurotic eyelid ptosis) that occurred during the study were reported in patients 65 years of age or older; none of these 3 events were assessed as related to study drug.

### Laboratory Assessments, Vital Signs, and ECG Assessments

None of the changes from baseline values for any hematology, chemistry, or urinalysis measure was particularly noteworthy. A total of 4060 serum samples were collected throughout the study and tested for the presence of general antibotulinum toxin antibodies. No patient shifted from negative at baseline to positive at any subsequent measure taken, including end of study/early termination. Accordingly, neutralizing antibody testing was not performed. One patient who had had previous exposure to botulinum toxin tested marginally positive (titer of 50) for the presence of antibotulinum toxin antibodies at the screening visit. This patient subsequently tested negative for the presence of antibotulinum toxin antibodies at all postbaseline visit measures. He also proved to be responsive to treatment. By IT day 2 and RT1 day 7, his GLS score at maximum frown had decreased to 0, from IT day 0 and RT1 day 0 scores of 3 and 2, respectively. 

None of the individual differences in the changes from baseline values were particularly noteworthy for any of the vital sign measures assessed. As summarized by the independent centralized electrocardiography facility, none of the ECG findings observed were of concern for the overall cardiac safety of prabotulinumtoxinA. Two patients had events that were considered to be clinically important for that patient: 1 had a new onset of complete right bundle branch block at the end of study visit, and another patient had atrial bigeminy at the IT day 30 visit that was resolved at the end of study visit.

### Efficacy

**Figure 3.**
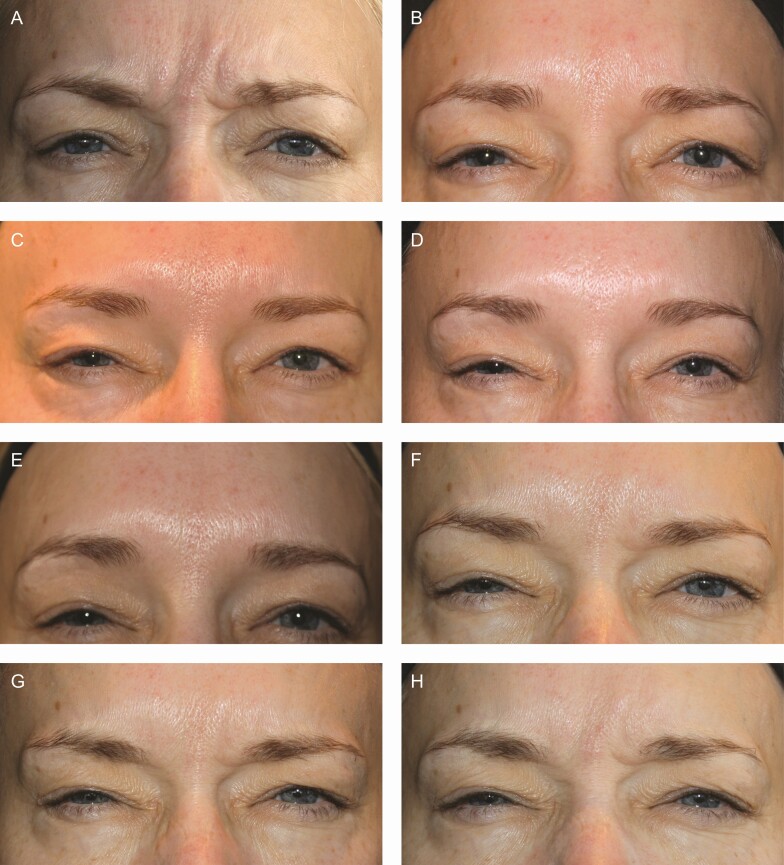
Photographs of glabellar lines at maximum frown at each of baseline (A), day 2 (B), day 7 (C), day 14 (D), day 30 (E), day 90 (F), day 120 (G), and day 150 (H) following initial treatment with 20 U prabotulinumtoxinA. This representative patient was a 48-year-old White female with Fitzpatrick skin type II and severe glabellar lines at maximum frown at baseline. She received 2 retreatments: the first at 5 months (ie, on day 150) and the second at 9 months (ie, on day 270) post baseline.

Representative photographs of a patient’s glabellar lines at maximum frown taken at baseline and at days 2, 7, 14, 30, 90, 120, and 150 are presented in Figure 3A-H.

The proportion of patients in the response-evaluable population with a ≥1-point improvement from baseline GLS score at rest on day 365 was the only efficacy endpoint for which 95% CIs were constructed. Patients who qualified for this analysis (436 by investigator assessment, 475 by patient assessment) were limited to those who completed the study who also had evidence of glabellar lines at rest at baseline (a baseline GLS score at rest of >0). Of these:

• 71.1% (66.6, 75.3) of patients had a ≥1-point improvement from baseline GLS score at rest on day 365 by investigator assessment
• 79.4% (75.4, 82.9) of patients had a ≥1-point improvement from baseline GLS score at rest on day 365 by patient assessment

**Figure 4.**
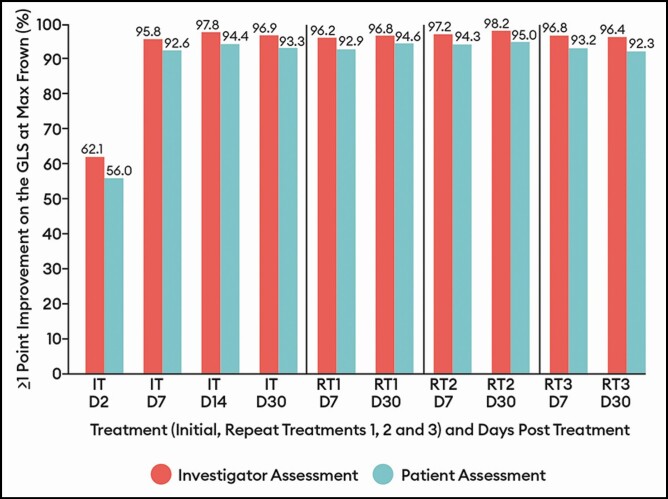
Percentage of patients with a decrease from baseline of ≥1 point on the Glabellar Line Scale at maximum frown by treatment at select visits: response-evaluable population. Efficacy assessments were not performed at repeat treatments D2 or D14. On and after day 90, patients were eligible for a repeat treatment if their Glabellar Line Scale score was ≥2 at maximum frown as judged by the investigator. If a patient did not have a Glabellar Line Scale score ≥2, they were followed monthly until eligible for repeat treatment or until the study ended on day 365. D, day; IT, initial treatment; RT, repeat treatment.

**Figure 5.**
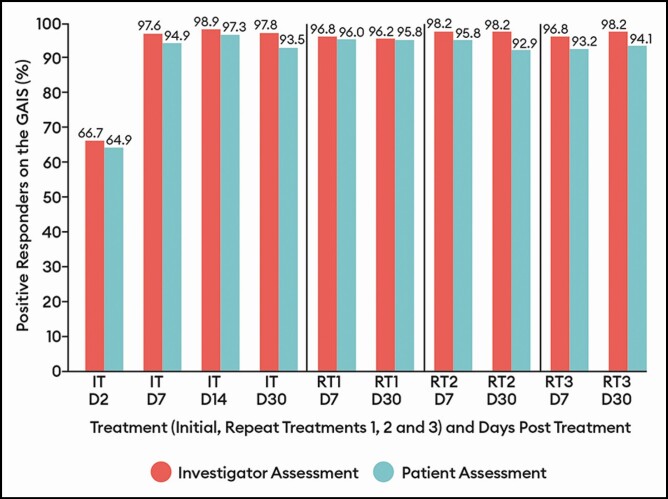
Percentage of patients with a positive response (improved/much improved) on the Global Aesthetic Improvement Scale by treatment at select visits: response-evaluable population. Efficacy assessments were not performed at repeat treatments D2 or D14. D, day; IT, initial treatment; RT, repeat treatment.

**Figure 6.**
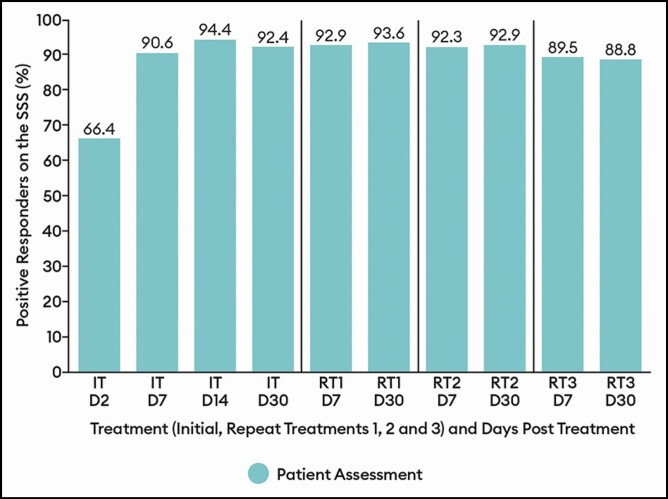
Percentage of patients with a positive response (satisfied/very satisfied) on the patient satisfaction scale by treatment at select visits: response-evaluable population. Efficacy assessments were not performed at repeat treatments D2 or D14. D, day; IT, initial treatment; RT, repeat treatment.

A marked response to treatment was evident from the first assessment day (day 2) following the IT by both investigator and patient assessment (Figures 4-6); on that day, 62.1% of patients by investigator assessment and 56.0% by patient assessment had achieved a ≥1-point improvement on the GLS at maximum frown. As illustrated in Figure 4, the percentage of patients with a ≥1-point improvement on the GLS at maximum frown peaked from the day 7 to day 30 visits for each treatment by both investigator assessment and by patient assessment. The percentages of patients with these outcomes at similar time intervals did not vary widely across RTs. For example, by investigator assessment and compared with 96.9% of patients at IT day 30, 96.8% at RT1 day 30, 98.2% at RT2 day 30, and 96.4% at RT3 day 30 experienced a ≥1-point improvement on the GLS at maximum frown (<2% absolute difference across treatments). A similar observation was noted for the percentage of patients with a ≥2-point improvement from baseline. That is, by investigator assessment and compared with 82.0% of patients at IT day 30, 81.2% at RT1 day 30, 82.9% at RT2 day 30, and 81.1% at RT3 day 30 experienced a ≥2-point improvement on the GLS at maximum frown (again, a <2% absolute difference across treatments, data not displayed). 

By both investigator and patient assessment, the percentage of patients with a positive response (ie, improved/much improved) on the GAIS showed little variation across treatments, ranging between 96.2% and 98.2% at the day 7 and day 30 visits for all treatments by investigator assessment and between 92.9% and 96.0% at the visits for all treatments by patient assessment (Figure 5). Similarly, the percentage of patients with a positive response (ie, satisfied/very satisfied) on the SSS did not vary widely across treatments, ranging between 88.8% and 93.6% at the day 7 and day 30 visits for all treatments (Figure 6).

## DISCUSSION

This repeat-dose study was conducted with the final commercial formulation of prabotulinumtoxinA. As such, the impact of repeat dosing on safety and efficacy was of particular interest. In this 570-patient, multicenter, open-label, 1-year phase II study, the safety of RTs of 20 U of DWP-450, up to a maximum total of 80 U, for the treatment of moderate to severe glabellar lines in adult patients was established based on a broad range of outcomes. Patients qualified for retreatment on and after day 90 only if their GLS score was ≥2 at maximum frown as assessed by the investigator. On average, patients qualified for and received 3 treatments. This was true for all patients as well as for the 487 patients who completed the 365 days of study and is particularly noteworthy given that 73.3% by investigator assessment and 81.2% by patient assessment had severe glabellar lines (GLS score = 3) at maximum frown at baseline. Among study completers, there was a slight trend towards longer retreatment periods. That is, for those who received 4 treatments, the mean sequential intervals between treatments were 93.9, 96.1, and 99.7 days (ranges of 77-145 days, 82-160 days and 65-154 days, respectively); for those who received 3 treatments, the mean sequential intervals were 130.6 and 137.0 days (ranges of 82-212 days and 84-217 days, respectively). This trend may be suggestive of a small cumulative benefit associated with RTs administered at intervals of 3 or more months. It may also be reflective of the fact that patients became eligible for a RT if they had a GLS score at maximum frown of at least moderate (≥2); it was not required that they return to their original baseline score. Importantly, there was no evidence of shortening retreatment periods that might otherwise have been suggestive of immunogenicity and/or the development of resistance.

The percentage of patients who experienced an AE after treatment did not increase with repeat exposure. In fact, the opposite trend was observed. While 41.2% of patients experienced 1 or more AEs over the course of this study, 25.3% experienced the event following the IT, representing 61.3% of all patients (144/235) who experienced an AE. Progressively lower percentages of patients experienced AEs following each RT. This trend was also observed for study drug–related AEs. These observations are typical of those reported for RTs of other botulinum toxins employed for this indication, including onabotulinumtoxinA, abobotulinumtoxinA (Dysport, Medicis Pharmaceutical Corp., Scottsdale, AZ), and incobutlinumtoxinA (Xeomin, Merz Pharmaceuticals GmbH, Frankfurt am Main, Germany); in all studies, the incidence of events was highest after the IT.^5-9^

Few patients (7/570, 1.2%) experienced a serious AE, none of which were study drug related, and few (16/570, 2.8%) patients experienced an AESI—of these, 9 experienced AESIs assessed as unrelated to study drug. All were mild in severity, none were assessed as severe or serious, and no one withdrew due to an AESI. Eight patients (1.4%) experienced a total of 11 AESI assessed as study drug related. All were categorized as eye disorders. Of particular interest, at 1.6%, the overall rate of patients with eyelid and/or eyebrow ptosis compared favorably to ptosis rates that have been reported for other toxins in other 12- to 13-month-long, repeat-dose studies, including 23 of 501 onabotulinumtoxinA-treated patients (4.6%)^5^ and 45 of 1200 abobotulinumtoxinA-treated patients (3.8%).^8^ In our study, 0.9% experienced a related eyelid ptosis event, 0.5% experienced a related eyebrow ptosis event, and 0.4% experienced an unrelated eyelid ptosis event.

There were no cases of seroconversion observed in this study. In over 4000 serum samples tested, only 1 patient (0.2%) tested marginally positive for the presence of antibotulinum toxin antibodies at the baseline visit only, and this patient had had previous exposure to botulinum toxin. No other patient tested positive for the presence of these antibodies. Similarly, no patient in either of the 2 US pivotal EV-001 and EV-002 studies (total of 492 prabotulinumtoxinA-treated patients), who was negative for the presence of botulinum toxin antibodies at baseline, tested positive at any of the repeat tests taken at days 30, 90 and end of study/early termination.^2^

None of the electrocardiographic findings observed were of concern for the overall cardiac safety of prabotulinumtoxinA. No other findings based on the laboratory hematology, chemistry, or urinalysis measures, vital signs, or utilization of concomitant medications was particularly noteworthy.

Although primarily a safety study, the efficacy of 20 U of prabotulinumtoxinA for the treatment of moderate to severe glabellar lines, up to a maximum of 4 treatments over the course of 1 year, was evident by all exploratory efficacy measures assessed. Utilizing each of the GLS at maximum frown, the GAIS, and the SSS, there was a similar pattern of rapid response to treatment in the first week posttreatment (as measured at IT day 2) with peak values observed at the IT day 14 visit and at RT day 7/day 30 visits. There was also no pattern of diminished response with RTs as would have been seen if, for example, an immunological response was mounting against the botulinum toxin. Similarly, no loss of effectiveness has been observed with RTs of other botulinum toxins approved for this indication.^5-9^

Because the effectiveness of a single treatment of 20 U prabotulinumtoxinA in treating glabellar lines at maximum frown had been established in the double-blind, placebo-controlled, phase III studies,^2,3^ it was of interest to utilize effectiveness data collected in this open-label phase II study to explore any treatment effect that repeated doses of toxin might have on glabellar lines at rest after 1 year. That is, if the underlying muscle remained relaxed for a period of 1 year, could there potentially have been remodeling of the soft tissue above the muscle in the glabellar region? Of interest, by study end, 71.1% of patients by investigator assessment and 79.4% by patient assessment who could potentially have experienced a ≥1-point improvement on the GLS at rest, did so. Further study is warranted to investigate this hypothesis.

In our study, a ≥1-point improvement from baseline on the GLS was the endpoint chosen to evaluate the change in glabellar lines over time. By design, a 1-point improvement on a 4-point scale of none, mild, moderate, and severe is intended to be clinically significant, both relevant to patients and meaningful to the physicians who treat them. Of note, as mandated by the FDA, a more statistically onerous primary efficacy endpoint was employed in the pivotal, single-dose, phase III studies: a ≥2-point improvement from baseline on the GLS at maximum frown at day 30 as independently agreed by both investigator and patient assessment.^2-4^ Although this high degree of correction is useful for regulatory purposes to establish the efficacy of a toxin for this indication, it is perhaps less clinically relevant to the treating physician and their patients who may prefer a more subtle, more natural-looking change. Of interest, by investigator assessment, between 81.0% and 82.9% of patients in our study achieved a ≥2-point improvement from baseline on the GLS at maximum frown at day 30 across RTs, as did between 81.5% and 86.1% of patients in the parallel repeat-dose EV-004 study.^10^ Similar outcomes were also achieved in each of the pivotal single-dose studies: by investigator assessment, 77.5% and 82.5% of prabotulinumtoxinA-treated patients in EV-001 and EV-002, respectively, experienced a ≥2-point improvement from baseline on the GLS at maximum frown at day 30; ^2^ in the EVB-003 study, 77.0% did (data on file). These data illustrate the consistency of response to 20 U prabotulinumtoxinA across all 5 studies of the clinical development program, regardless of whether the studies had an open-label or double-blinded controlled design.

One clear limitation of this study is its open-label (ie, non-blinded), non-randomized, uncontrolled design. As a consequence, all efficacy endpoints were considered exploratory in nature; a blinded independent assessment of efficacy endpoints was not performed. Still, within the confines of a clinical study program, it is the design that best approximates the expected utilization of the product by the general population in the real world. With the rigorous monitoring of safety measures, it was considered suitable for the collection of long-term safety data related to repeat dosing of a botulinum toxin for this indication. A second limitation, also seen in the placebo-controlled studies,^2,3^ is a reflection of the current clinical profile reported for this product^11,12^ in which males, patients 65 years of age and older, and those with skin of color are under-represented; 89.5% of our study patients were female, 91.1% were less than 65, and 76% were identified as White. Similar disparities are also evident in the patient populations of the long-term studies with other toxins.^5-9^

## CONCLUSIONS

In summary, the safety of RTs of 20 U of prabotulinumtoxinA for moderate to severe glabellar lines in adult patients was established in this multicenter, open label, long-term, phase II study based on a broad range of outcomes, including AEs and serum antibody testing. Of key importance to clinicians, progressively lower percentages of patients experienced study drug–related AEs with RTs. Furthermore, an examination of exploratory efficacy outcomes suggests that there is no pattern of diminished effectiveness with RTs.

By its nature, an open label, repeat-dose, phase II study such as this reflects real-world clinical practice and provides visibility on what happens following exposure to RTs. As such, it complements findings based on the single-dose, phase III studies on which the safety and efficacy of a toxin is first established. Importantly, many of the safety and efficacy outcomes evaluated in our studies (eg, AEs such as headache and ptosis, and efficacy measures such as glabellar line diminishment, overall aesthetic improvement, and patient satisfaction) are inherently relevant to the clinical practice of physicians who administer botulinum toxin injections and to their patients who seek out this type of treatment.
