# Corrigendum to: The Second of Two One-Year, Multicenter, Open-Label, Repeat-Dose, Phase II Safety Studies of PrabotulinumtoxinA for the Treatment of Moderate to Severe Glabellar Lines in Adult Patients

**DOI:** 10.1093/asj/sjab230

**Published:** 2021-06-30

**Authors:** Z Paul Lorenc, Jeffrey M Adelglass, Rui L Avelar, Leslie Baumann, Kenneth R Beer, Joel L Cohen, Sue Ellen Cox, Steven H Dayan, Jeffrey S Dover, Jeanine B Downie, Zoe Diana Draelos, Mitchel P Goldman, John E Gross, John H Joseph, Joely Kaufman-Janette, Ronald L Moy, Mark Nestor, Joel Schlessinger, Stacy R Smith, Robert A Weiss

In the article “The Second of Two One-Year, Multicenter, Open-Label, Repeat-Dose, Phase II Safety Studies of PrabotulinumtoxinA for the Treatment of Moderate to Severe Glabellar Lines in Adult Patients” by Lorenc et al in *Aesthet Surg J.*, sjaa382, https://doi.org/10.1093/asj/sjaa382, in the originally published version of this manuscript, errors were noted in co-author Joel Schlessinger’s name, affiliation, and disclosure statement. This corrigendum corrects errors in the previously published version.

In the author list and affiliations, Joel Schlessinger’s full name should read: “Joel Schlessinger” and his affiliation should read: “Dr Schlessinger is a dermatologist in private practice, Omaha, NE, USA.”

The Disclosure section should read: “Dr Schlessinger is a shareholder of Evolus, Inc., (Newport Beach, CA) and has served on the advisory board; he is also a shareholder of AbbVie Inc. (North Chicago, IL), and has served as a researcher for Merz (Frankfurt, Germany), Galderma (Lausanne, Switzerland), Allergan (Irvine, CA)/AbbVie (North Chicago, IL) and Croma Pharma (Leobendorf, Austria)”. The author regrets these errors.

